# Congenital isolated adrenocorticotropic hormone deficiency in a newborn caused by *TBX19* mutation: a case report and literature review

**DOI:** 10.3389/fped.2024.1493387

**Published:** 2024-11-27

**Authors:** Yinxia Dang, Juanli Zhang, Fan Wang

**Affiliations:** Department of Neonatology, Lanzhou University Second Hospital, Lanzhou, China

**Keywords:** TBX19, congenital isolated adrenocorticotropic hormone deficiency, neonatal, hydrocortisone, prognosis

## Abstract

**Background:**

To investigate the clinical phenotype, genetic characteristics, and prognosis of isolated adrenocorticotropic hormone deficiency in a newborn (IAD, OMIM 201400) caused by mutation of the *TBX19* gene.

**Case presentation:**

The clinical features, diagnosis, treatment, and prognosis of a newborn with IAD admitted to our hospital were retrospectively analyzed. The patient and his parents were also examined by whole exome sequencing. We used the terms “newborn”, “child”, “congenital isolated adrenocorticotropic hormone deficiency”, and “TBX19” to retrieve relevant studies published up to December 2023 from the following databases: China National Knowledge Infrastructure (CNKI), Wanfang Database, Chinese Medical Journal Full-text Database, VIP database, Sinomed, PubMed, Embase, and Web of Science. The clinical and genetic characteristics of children from these other publications were summarized. The newborn boy with IAD was admitted to our hospital with poor mental response, feeding difficulties, hypoglycemia, and jaundice. The brain and adrenal MRI results were normal. Clinical whole exome sequencing showed that the boy carried compound heterozygous variants in the *TBX19* gene. Specifically, the first exon had a novel frameshift mutation, c.240-246del(p.leu81Profs*54, NM_005149.3), and a missense mutation, c.377C>T(p.Pro126leu, NM_005149.3). The literature search found 34 additional cases from 4 Chinese-language articles and 12 English-language articles. The main clinical manifestations were hypoglycemia, jaundice, convulsions, feeding difficulties, poor mental response, hypotonia, and growth retardation. There were 24 cases with *TBX19* mutations, and 19 different mutation sites. Among the 15 patients with different degrees of nervous system developmental delays, 13 initiated treatment when more than 1-year-old.

**Conclusion:**

IAD from *TBX19* mutation causes nonspecific symptoms. Genetic testing is the key to diagnosis. Early diagnosis and treatment can help to improve the prognosis and prevent neurological complications.

**Clinical Trial Registration:**

identifier (2024A-796).

## 1 Introduction

Isolated adrenocorticotropic hormone deficiency (IAD, OMIM 201400) is a rare endocrine disorder that is often hereditary. It is characterized by a deficiency of adrenocorticotropic hormone (ACTH), resulting in decreased production of adrenal cortisol but normal production of other pituitary hormones. Neonatal IAD can cause health disorders, such as long-term cholestatic jaundice, hypoglycemia, epilepsy, feeding difficulties, slow reaction time, weight loss, lethargy, and coma, and it can be life-threatening in severe cases (1). At present, the exact etiology of IAD is not fully understood, but some studies showed that it may be related to genetic and innate factors, especially mutation of the *TBX19* gene, which encodes a T-box family transcription factor (TPIT) that regulates the synthesis and secretion of ACTH. TBX19 occurs in the anterior pituitary gland cells and is regulates the expression of proopioid- melanocortin (POMC), a precursor of ACTH (2). Studies have found that up to 65% of neonatal IAD cases have *TBX19* mutations (3), and that approximately 20% of these neonates will die without prompt diagnosis and treatment deaths (4).

This article reports a case of neonatal IAD, and describes the characteristics of this disease by analysis of clinical manifestations, hormone levels, and laboratory tests. Gene sequencing confirmed the presence of a *TBX19* mutation. IAD can cause a variety of non-specific clinical symptoms, such as hypoglycemia, jaundice, convulsions, feeding difficulties, growth retardation, and this can lead to delays in diagnosis. However, rapid diagnosis is essential for early treatment. This study deepened the understanding of IAD by thorough analysis of a case and a literature review, and emphasized the importance of early diagnosis for improved prognosis.

## 2 Case presentation

### 2.1 Medical history of the patient

A 7-day-old male was transferred to our hospital due to “refusal of milk with poor response for 4 days”. The patient's mother (gravida 2, parity 2), gave birth by cesarean section. The newborn had a birth weight of 3,000 g, a length of 50 cm, and had no history of asphyxia. On the third day after birth, the infant presented with milk refusal, poor mental response, yellow staining of the skin and mucosa of the whole body, and fever (38.4 ℃), but no convulsions. The local hospital diagnosed “neonatal septicemia and jaundice”. After management of symptoms for 4 days, his body temperature returned to normal, but he still had poor mental response, and was transferred to the Department of Neonatology of our hospital. At admission to our department, his blood glucose level was 2.02 mmol/L, and he was discharged after 12 days of supportive treatment. On the 28th day after birth, the jaundiced skin and mucosa recurred, although we did not measure the blood glucose level. At this time, the results from whole exome sequencing (WES) were available, and he was readmitted for 7 days, and received regular follow-up in the outpatient clinic.

### 2.2 Medical history of the parents

The father was in good health with blood type A, the mother had blood type B, and the marriage was non-consanguineous. The mother had a history of hypothyroidism during pregnancy for which she received levothyroxine sodium tablets. Their first child was a girl who unfortunately passed away during the neonatal period from unknown causes.

### 2.3 Physical examination of the patient

When the neonate was first admitted to our department (age of 7 days), his body temperature was 36.8 ℃, pulse rate was 160 beats per minute, respiration rate was 40 breaths per minute, and blood pressure was 85/51 mmHg (11/6.8 kPa). At that time, his length was 50 cm and his weight was 3,150 g. The patient had a relatively sluggish response to stimulation and emitted a weak cry. No seizures or signs of impaired consciousness were observed. The skin had a yellowish hue throughout, with an absence of pigmentation. The scleral areas were also yellowish, and the pupil diameter was 2 mm, with responsive light reflexes. Furthermore, both lungs displayed coarse breath sounds upon auscultation, palpation of the liver approximately 1 cm below the subcostal margin indicated no splenomegaly, and there was also no umbilical hernia. However, there was varus deformity of the left foot and weak muscle tone in all extremities. Primitive reflexes were elicitable.

### 2.4 Auxiliary examinations

Laboratory tests performed at the same time indicated a blood glucose level of 2.02 mmol/L, serum sodium level of 130.6 mmol/L, total bilirubin level of 194.5 µmol/L, direct bilirubin level of 24.3 µmol/L, indirect bilirubin level of 170.2 µmol/L, γ-glutamyl transpeptidase (GGT) level of 235 U/L, and bile acid level of 134.7 µmol/L. The procalcitonin (PCT) level was 0.105 ng/ml, but a blood culture and sputum culture were both negative for infection. Following supportive treatment, the PCT level returned to normal and the mental response improved, although milk refusal, hypoglycemia, and jaundice persisted without explanation, perhaps suggesting neonatal sepsis. Further endocrine examination showed low levels of cortisol (<0.1 µg/dl) and adrenocorticotropic hormone (ACTH, 2.l pg/ml), but the levels of growth hormone (GH), luteinizing hormone, and thyroid stimulating hormone were within normal limits (Table 1). The low score on the Neonatal Behavioral Neurological Assessment (NBNA) indicated impaired behavioral responses. The NBNA evaluates newborn behavioral capabilities, passive and active muscle tone, primitive reflexes, and general condition. The scale includes 20 items, scored out of a total of 40 points. A score below 35 is considered abnormal, the subject in question attained a score of 24.

**Table 1:** Laboratory test results of the child from admission to the last follow-up.

Parameter	Age (days)							Reference range
	7	31	37	48	64	100	135	
ACTH	2.1	1.1	–	2.8	<5	2.9	3.3	7.2–63.3 pg/ml
Cortisol	<1.0	<0.1	1.6	<1.0	–	<1.0	<1.0	4.8–19.5 µg/dl
Blood glucose	2.02	2.70	4.10	3.80	4.20	4.60	4.17	3.90–6.10 mmol/L
Total bilirubin	218.2	227.6	177.5	91.3	26.6	7.1	5.6	<21 µmol/L
Direct bilirubin	23.6	33.7	30.5	18.7	7.2	1.9	1.6	<4 µmol/L
Indirect bilirubin	194.6	193.9	147.0	72.6	19.4	5.2	4.0	0–22.1 µmol/L
Total bile acids	–	134.7	112.0	104.0	20.6	5.9	6.2	<10 µmol/L
GGT	241.0	430	317	162	63	33	29	9–150 U/L
Hemoglobin	134	106	88	99	–	122	144	97–183 g/L
AST	20	26	23	32	52	43	38	21–80 U/L
ALT	5	8	10	15	39	29	33	8–7 1 U/L
Na	130.6	131.0	131.7	134.9	133.9	134.9	–	135–150 mmol/L
Urinary glucose	N	N	–	–	–	–	–	N
Urinary ketones	N	N	–	–	–	–	–	N
Free T3	5.25	–	–	–	–	–	–	2.49–7.10 pmol/L
Free T4	14.01	–	–	–	–	–	–	12.00–29.34 pmol/L
TSH	8.067	–	–	–	–	–	–	0.47–12.25 pmol/L
Growth hormone	8.72	–	–	–	–	–	–	0.06–5.00 ng/ml
IGF-1	92.2	–	–	–	–	–	–	18–172.00 ng/ml
Procalcitonin	0.105	–	–	–	–	–	–	0.000–0.046 ng/ml

ACTH, adrenocorticotropic hormone; TSH, thyroid stimulating hormone; AST, aspartate aminotransferase; ALT, alanine aminotransferase; IGF-1, insulin-like growth factor 1; GGT, γ-glutamyl transpeptidase; N, normal; –, not determined.

**Figure 1:**
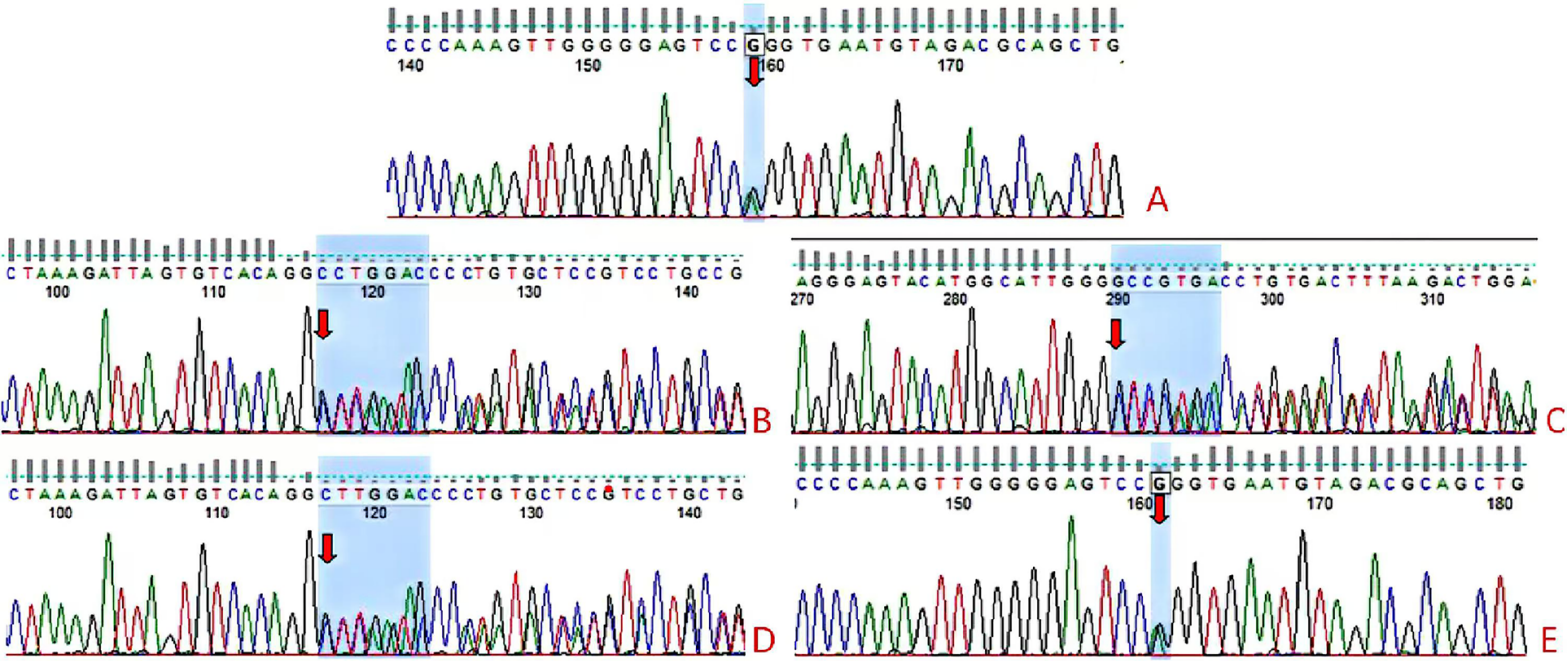
*TBX19* gene sequencing results of the child and parents. The 377C>T(p.Pro126leu) and C.240-246del(p.leu81Profs*54) compound heterozygous variants were identified in the child **(A–C)**. The mother had a heterozygous variant c.240-246del(p.leu81Profs*54) **(D)** and the father had the heterozygous variant 377C>T(p.Pro126leu) **(E)**.

On day 31 after birth, cranial magnetic resonance imaging (MRI), pituitary MRI, and adrenal CT all yielded normal results. However, an electroencephalogram (EEG) was abnormal, with a slow background rhythm. We analyzed the WES results to search for variants that may be associated with the clinical phenotype, and also performed genetic analysis of the parents. The patient had compound heterozygous variants, c240-246del(p.leu81Profs*54, NM_005149.3) and c377C>T(p.Pro126leu, NM_005149.3), that were in exon 1 of the *TBX19* gene, and these variants were inherited from the father and mother (Figure 1). According to the guidelines of the American College of Medical Genetics, p.Leu81profs*54 is a suspected pathogenic variant, although the clinical significance of p.Pro126leu is unknown. We initiated hydrocortisone (HC) replacement therapy when the infant was 31-days-old, after which the levels of bilirubin and bile acid gradually decreased, and the levels of ACTH and cortisol gradually increased (Table 1).

### 2.5 Treatments prior to diagnosis

During the whole hospitalization, the parents of the child obtained informed consent and actively cooperated with the treatment. When the child was admitted to the hospital for the first time, we considered neonatal hypoglycemia, neonatal hyperbilirubinemia and neonatal sepsis based on symptoms, physical signs and auxiliary examinations. We thought the possible cause of neonatal hypoglycemia might be endocrine disorders. A normal head MRI excluded endocrine dysfunctions caused by the pituitary gland and hypothalamus. We took appropriate temporary treatment measures. We maintained stable blood sugar by intravenous infusion of 10% glucose, conducted intermittent phototherapy and used ursodeoxycholic acid capsules for liver protection and choleresis. At the same time, we used piperacillin sodium and tazobactam sodium for injection to treat neonatal sepsis for seven days. After treatment, the child's clinical symptoms disappeared and there was no recurrence of hypoglycemia. The child was discharged after improvement.

The second time, the child had hypoglycemia and jaundice symptoms again. At this time, as we had previously suspected neonatal endocrine diseases and had the report of whole exome sequencing (WES), the child was clearly diagnosed with neonatal IAD (age of 31 days). We immediately administered HC (10 mg/m^2^/day) for replacement therapy. After the condition stabilized, the child was discharged. We instructed the child to take medicine on time and regularly monitor blood sugar, adrenal cortical hormones, cortisol, etc. at the outpatient clinic to adjust the dosage.

### 2.6 Treatments after the diagnosis

**Figure 2:**
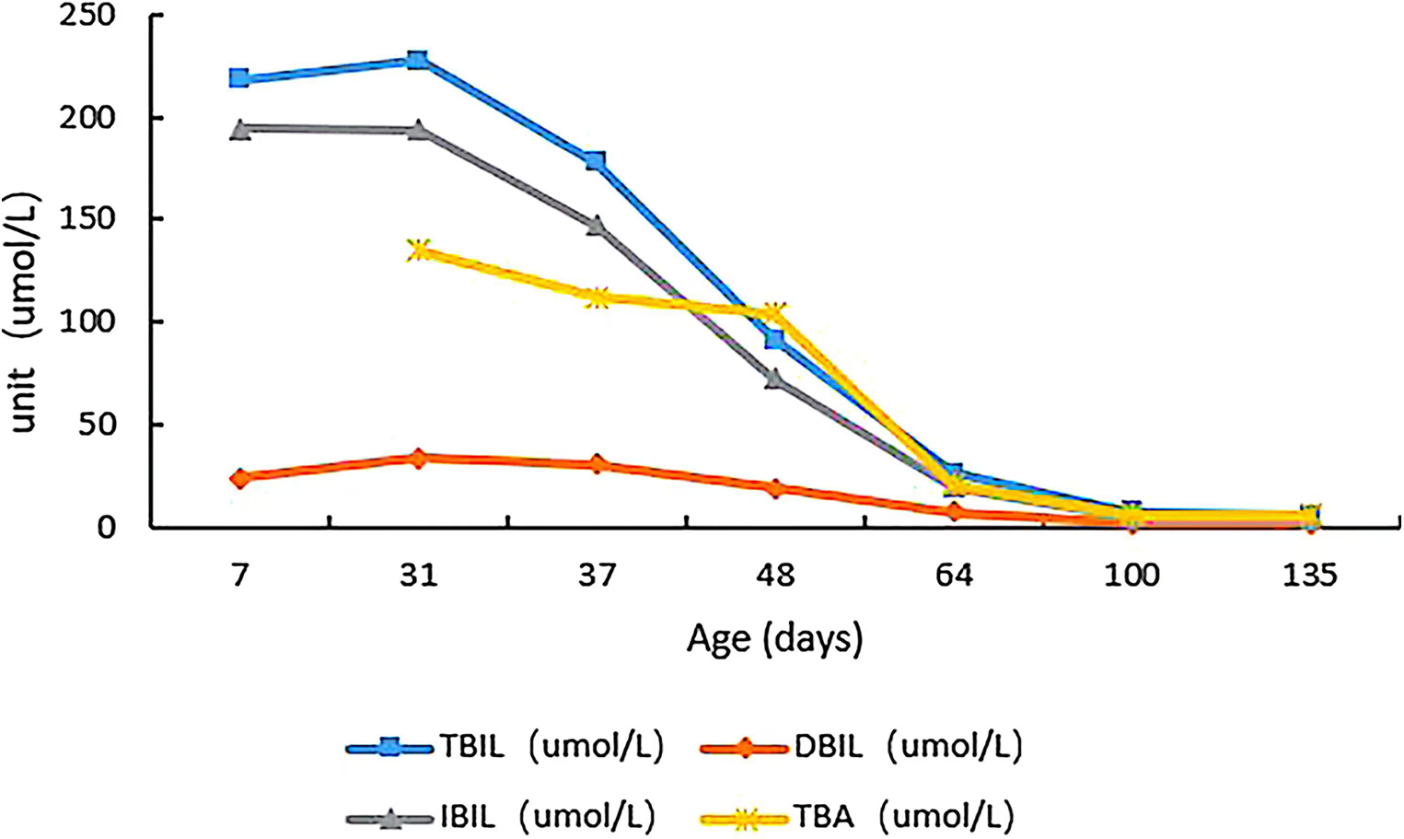
Changes in total bile acids, total bilirubin, direct bilirubin, and indirect bilirubin of the child from admission to the last follow-up.

We initiated the HC treatment (20 mg/m^2^/day) immediately after receiving the WES results. During the follow-up, we also administered two cycles of intermittent phototherapy, and this led to a relief from jaundice, and decreases in the levels of total bile acids and bilirubin (Figure 2). Additionally, the blood glucose levels increased to the normal range, and there was no evidence of liver disease, leading to patient discharge.

**Figure 3:**
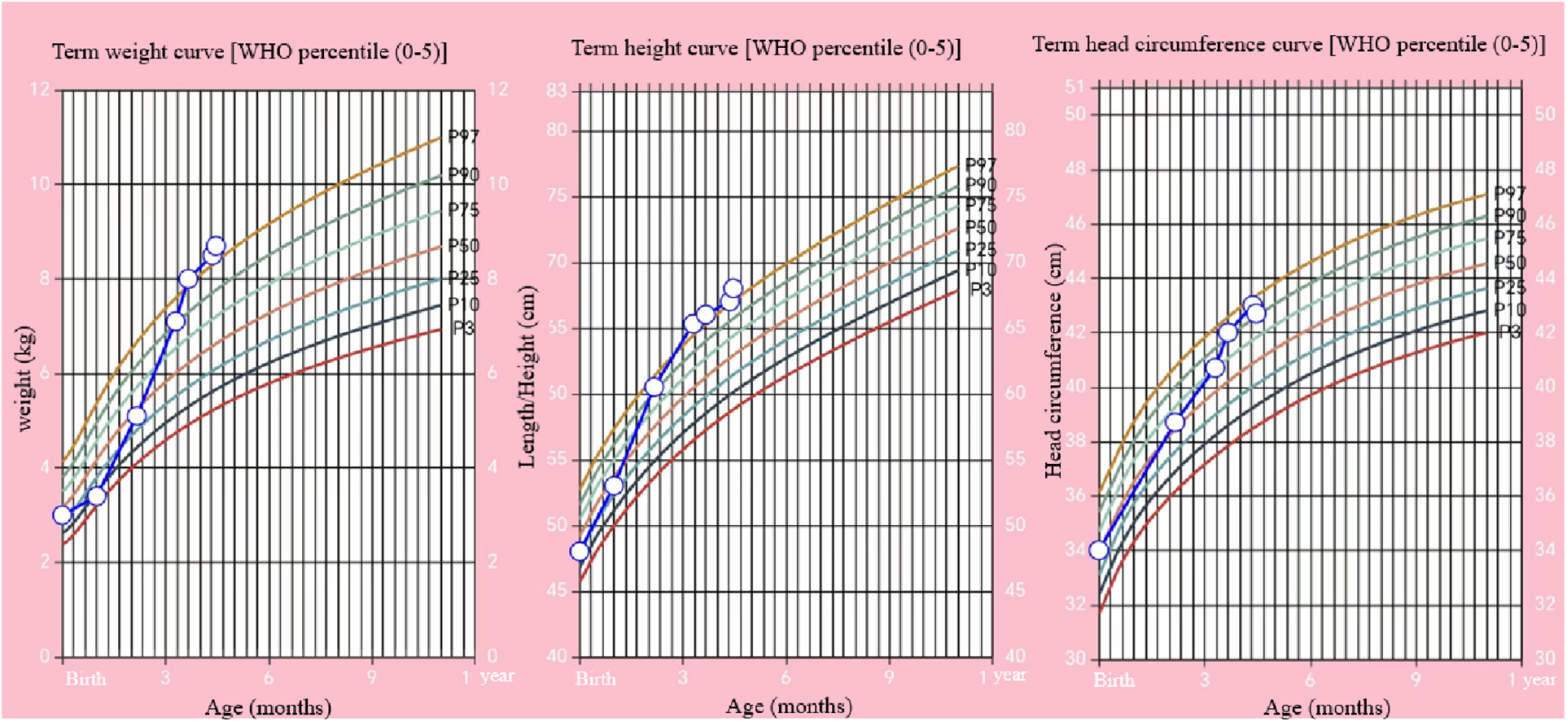
Changes of body weight, length, and head circumference of the child from birth to 135 days (open circles) and WHO percentiles.

During the 100-day follow-up period, we gradually decreased the HC dosage to 10 mg/m^2^/day. At the last follow-up (135 days after birth), the patient's height was 68 cm (>P97), weight was 8.7 kg (>P97), head circumference was 42.7 cm (P90–P97), and he appeared to have normal mental development (Figure 3). The ACTH and cortisol levels gradually increased, but did not fully return to normal, although the bilirubin level showed gradual recovery (Table 1). On day 100 of the HC treatment, the bilirubin level returned to normal range (Table 1; Figure 2). Moreover, the child's blood glucose remained stable, he had no clinical symptoms or adrenal crisis, and his liver function remained within normal limits.

## 3 Literature analysis

### 3.1 Information retrieval

For a literature search, we used the terms “newborn”, “child”, “congenital isolated adrenocorticotropic hormone deficiency”, and “TBX19” to retrieve relevant studies until December 2023 from the following databases: China National Knowledge Infrastructure (CNKI), Wanfang Database, Chinese Medical Journal Full-text Database, VIP database, Sinomed, PubMed, Embase, and Web of Science. This search of case reports of IAD in any language led to identification of 35 cases, including the current infant. Twelve studies were in the English language (3–14) and 4 studies were in the Chinese language (15–18) (Supplementary Material).

### 3.2 General information

There were 24 boys (69%) and 11 girls (31%). There were 24 newborns (69%), 7 cases (20%) between 1-month and 3-years-old, and 4 cases (11%) over 3-years-old (Supplementary Material). Further analysis of the age at which IAD treatment began showed that 16 cases (46%) were between 0 and 3-months-old, 3 cases (8%) were between 3-months-old and 1-year-old, 7 cases (20%) were between 1-year-old and 3-years-old, and 9 cases (26%) were more than 3-years-old. The most common clinical symptoms were hypoglycemia (*n* = 33), jaundice (*n* = 21), and epilepsy and convulsion (*n* = 18). The other notable symptoms were feeding difficulties and poor mental response (*n* = 7), recurrent respiratory tract infection (*n* = 2), abdominal pain and vomiting (*n* = 6), and hypothermia (*n* = 2). The laboratory data showed that all 35 patients had low levels of ACTH and cortisol; 33 cases had blood glucose levels lower than 2.8 mmol/L and 2 cases had unknown blood glucose levels without hypoglycemia symptoms; 17 cases had increased total bilirubin levels, 1 case had a normal level, and 17 cases had unknown levels; 14 cases had hyponatremia, 11 cases had normal serum sodium levels, and 10 cases had unknown levels. The MRI results showed that 29 cases had normal pituitary results, 3 cases had varying degrees of pituitary changes, and 3 cases had unknown results; the brain MRI results showed bilateral subdural hematoma, hypoglycemic encephalopathy, and left cerebral hemisphere diffuse atrophy (1 case each). Among all 35 children, 7 were from consanguineous marriages, and 16 children were from families that had other children who had a disease or died of unknown causes.

### 3.3 Methods of diagnosis

Whole genome sequencing was performed in all 35 children. Twenty-four children had *TBX19* gene mutations (with mutations at 19 different sites), 9 had no *TBX19* mutations, and the results of the other 2 cases were not reported.

### 3.4 Treatment

Thirty-five patients received long-term HC replacement therapy after the diagnosis of IAD. After HC treatment, the clinical symptoms completely resolved in 30 patients, the clinical symptoms of 3 patients were significantly resolved, except for refractory seizures that were treated with antiepileptic therapy, and the effects of treatment in 2 patients were not reported.

### 3.5 Complications and prognosis

After treatment, 18 cases had no neurological development delay, 15 cases had different degrees of neurological development delays, and follow-up data of 2 cases were not reported. Among the 15 cases with complications, 13 cases began treatment after the age of 1 year, and 1 case did not receive regular HC treatment (Supplementary Material).

## 4 Discussion

We described a newborn who was admitted due to milk refusal and poor mental response. Our initial analysis led to identification of hypoglycemia, hyponatremia, and cholestatic jaundice. Clinical tests showed abnormally low levels of ACTH and cortisol, but normal levels of other pituitar*y* axis hormones, such as growth hormone and thyroid hormone. MRI indicated a normal pituitary gland, and CT indicated a normal adrenal gland. All of these observations led to a suspicion of IAD. Further whole exome capture and sequencing were performed on the genomic DNA of the examinee. The obtained data had an average sequencing depth of at least 90X in the known exon and upstream and downstream 5 bp sequences of human genome genes, and about 98% of the target sequences had a sequencing depth of more than 20X. Base recognition was performed on all sequenced fragments. The detection mainly used the GATK software suite for sequencing data analysis. The sequenced fragments were compared with the UCSC hg19 reference genome through BWA. According to the ACMG guidelines, WES of the infant led to the identification of two variants in the *TBX19* gene, a novel frameshift mutation (c.240-246del p.leu81Profs*54, NM_005149.3) and a missense mutation (c.377C>T p.Pro126leu, NM_005149.3), each of which could lead to dysfunctional production of ACTH. The first mutation may lead to premature termination of the coding sequence, and the subsequent loss of functional TBX19 protein can lead to IAD. The second mutation was previously reported in 4 children with IAD from two different families (6, 18). CIAD caused by a dysfunctional *TBX19* gene has autosomal recessive inheritance, and the two variants in our patient were likely responsible for dysfunction of the TBX19 protein, and the patient's decreased production of ACTH (13, 16).

A 2012 case series examined 91 patients with IAD: 37 neonates with *TBX19* mutations, 32 neonates without *TBX19* mutations, and 22 juveniles without *TBX19* mutations (2). All neonates with *TBX19* mutations developed neonatal hypoglycemia and had complete IAD, but the incidences of these and other clinical signs (e.g., long-term cholestatic jaundice and seizures) were lower in the two groups without *TBX19* mutations. Consistent with these results, 24 of the 35 neonates in our study who had CIAD and *TBX19* mutations had manifestations of hypoglycemia, jaundice, convulsions, feeding difficulties, and growth retardation (mostly during the neonatal period), 2 cases had recurrent upper respiratory tract infection, and 1 case had sepsis (13, 17, 18). The auxiliary examinations showed that all 35 cases had low ACTH, low cortisol, high bilirubin, and low serum sodium, and that 2 of the 35 cases had life-threatening adrenal crisis (9, 18). The main treatment for IAD is replacement with long-term exogenous HC and anti-epileptic treatment for cases with seizures. Early diagnosis and prompt treatment usually lead to good prognosis, and delayed treatment may lead to serious metabolic disorders and an increased risk of mortality (19). The symptoms and laboratory indexes of the 35 IAD children we examined mostly resolved after HC replacement therapy;18 of them had no delays in neurological development and 15 had varying degrees of delays in neurological development. Among the 15 children with complications, 13 began treatment after the age of 1 year, and 1 case did not receive regular HC treatment. This suggests a need for individualization of the HC regimen for patients with IAD. In cases of disease, injury, surgery, or other major stress, the dose of HC should be adjusted in a timely manner, and long-term follow-up should be conducted. This is particularly important to ensure the long-term health and normal development of these children (19).

The symptoms of neonatal CIAD are nonspecific, so a differential diagnosis is needed to exclude other diseases, such as congenital adrenal hyperplasia (CAH), congenital hypopituitarism, familial glucocorticoid deficiency (FGD), as well as mutations in genes for the ACTH receptor or genes related to ACTH synthesis, and especially mutations in genes responsible for FGD, a rare autosomal recessive disorder characterized by hyperpigmentation of the skin and mucosa, severe hypoglycemia, occasional seizures and coma, feeding difficulties, growth retardation, and infections. In clinical practice, it is necessary to consider the combination of clinical manifestations, hormone levels, results from genetic testing, and imaging results to establish a diagnosis of CIAD (20).

The neonate carries compound heterozygous variants of the TBX19 gene (c.240_246del (p.Leu81Profs*54 NM_005149.3) and c.377C>T (p.Pro126Leu NM_005149.3) from the mother and father respectively. When the parents have another child, the probability of inheriting both abnormal genes is 1/2 for each, and the overall probability of carrying both simultaneously is 25%. The WES results of this neonate showed no gene sequence heterogeneity within the tested genomic range. All gene alleles in the sample were consistent, with no evidence of chimera formation. In reported cases, studies mostly determined pathogenic mutations and inheritance patterns (autosomal recessive) through patient genetic testing. Analyses of IAD patient families found TBX19 mutations mainly as homozygous or compound heterozygous, with no evidence of chimera in the same patient. Most results fit the traditional autosomal recessive model. However, due to testing technique limitations and sample size, the possibility of TBX19 gene mutation chimera in some cases or individuals cannot be excluded. Future research may need to improve testing precision, expand sample size, and use advanced methods to detect chimera and its impact on the disease for better guidance. In conclusion, neonatal CIAD is a rare and severe disease that has nonspecific clinical symptoms, making misdiagnosis or missed diagnosis likely. The wide application of genetic testing technologies and the increasing number of CIAD case reports worldwide have improved awareness of this disease, and medical staff are now better positioned to make the most appropriate interventions to decrease the risk of long-term complications in these patients. The successful management of CIAD must consist of early diagnosis, individualized treatment, and long-term follow-up to ensure the best growth and development outcomes.

## Data Availability

The datasets presented in this study can be found in online repositories. The names of the repository/repositories and accession number(s) can be found in the article/Supplementary Material.
