# Supplementary material for: Congenital isolated adrenocorticotropic hormone deficiency in a newborn caused by TBX19 mutation: a case report and literature review
Source: Front Pediatr. 2024 Nov 27;12:1493387. doi: 10.3389/fped.2024.1493387 (PMC11631573; doi:10.3389/fped.2024.1493387)
Supplement: Supplementary file 1 [file Table1.doc]

**Supplementary Table 1. Characteristics of 35 children with isolated adrenocorticotropic hormone deficiency.**

| **Patient #** | **Gender** | **Age at onset** | **Age at treatment initiation** | **Consanguineous parents** | **Other children affected** | **Symptoms at onset** | **Cortisol, µg/dL** | **ACTH, pg/mL** | **Blood glucose, mmol/L** | **TBIL, umol/L** | **DBIL, umol/L** | **Na, mmol/L** | **Pituitary MRI** | ***TBX19* mutation and sites** | **Age & prognosis at follow-up** | **Delay in development** | **Reference** |
| --- | --- | --- | --- | --- | --- | --- | --- | --- | --- | --- | --- | --- | --- | --- | --- | --- | --- |
| 1 | M | 5 d | 2.6 yr | – | – | h, j, c, p | <1.0 | 1.5 | 1.3 | 316.6 | 12.7 | 140.7 | Normal | He mutations: c.205C>T(p.R69W), fragment deletion: (g.168,247,374-g.168,278,264) | No symptoms after 1 yr and 8 m of treatment | N | [5] |
| 2 | F | 2 d | 52 d | – | Y | h, j, c | 0.23 | <1.0 | 0.7 | 358.5 | 27.1 | 124.0 | Normal | Ho mutation: C.377(exon2)C>T,p.P126L | 2 yr, no flare of symptoms | N | [6] |
| 3 | M | 1 d | 2 m | – | Y | h, j, c | low | <1.0 | 1.1 | 232.0 | 15.9 | 126.0 | Bilateral subdural hematomas | Ho mutation: C.377(exon2)C>T,p.P126L | 7 yr, still had intractable seizures | Y | [6] |
| 4 | M | 1 d | 15 d | – | Y | h, j, p | 0.08 | 2.32 | 1.7 | 361.0 | 0 | 138.0 | Normal | He mutations: c.122T>G(a) and c.787C>T(b) | 5 m, no symptoms | N | [15] |
| 5 | M | 7 d | 3 m | Y | – | h, j | 0.1 | 0.08 | <2.4 | 210.0 | 160 | – | Normal | – | Unknown follow-up and prognosis | – | [7] |
| 6 | M | 7 d | 3m | Y | – | h, j | 0.14 | 0.9 | <2.4 | 166.0 | 137 | – | Normal hyperintensity of adenohypophysis disappeared | – | Unknown follow-up and prognosis | – | [7] |
| 7 | F | 1 d | 2m | – | Y | h, j, c | 1.2 | 1 | 2.1 | 102.0 | 73.1 | 136.0 | – | Ho mutation: c.856 C>T(p.R286*) | 5 yr, hypoglycemic episodes were significantly reduced | Y | [8] |
| 8 | M | 1 d | 7d | – | Y | h, j, p | 0.05 | <5 | 2.6 | 161.5 | 13.6 | 136.0 | – | Ho mutation: c.856 C>T(p.R286*) | After 10 m of treatment, no symptoms | N | [8] |
| 9 | M | 1 d | 5m | Y | N | h, j, low muscle tone | <1.0 | <0.4 | low | – | – | low | Normal | Ho mutation: c.840del (pGlu280Asp fs*27) | 3 yr, no symptoms | N | [9] |
| 10 | M | 1 d | 2m | Y | Y | h, j, low muscle tone | <0.11 | <0.2 | 1.1 | – | – | low | Moderate hypoplasia of anterior pituitary | Ho mutation: c.840del (pGlu280Asp fs*27) | 5 yr, no symptoms | N | [9] |
| 11 | F | 1 d | 6d | Y | Y | h | <1.0 | <0.4 | 0.6 | – | – | – | Normal | Ho mutation: c.840del (pGlu280Asp fs*27) | 7 yr, three episodes of adrenal crisis with hypoglycemia | N | [9] |
| 12 | M | 1 d | 10d | Y | Y | p, low muscle tone | <0.11 | 0.6 | – | – | – | 130.0 | – | Ho mutation: c.840del (pGlu280Asp fs*27) | 1 yr, no symptoms | N | [9] |
| 13 | M | 1 d | 11d | – | – | h, j, p, apnea | <0.2 | low | 1.1 | 306.0 | 40.8 | – | Normal | He mutations: c.158_159 delGA and c.535C>T | 20 m, no symptoms | N | [4] |
| 14 | M | 1 d | 13m | – | Y | h, j, c, p | 1.45 | 4.8 | 0.13 | – | – | 125.8 | Normal | He mutations: c.535C>T(p.R179X) and c.298C>T(p.R100C) | No symptoms | Y | [16] |
| 15 | F | 1 d | 2m | – | – | h, j, c | 0.054 | 1.3 | 0.4 | high | – | 138.0 | Normal | Ho mutation: c.856C>T (p.R286*) | 14.6 yr, no symptoms | Y | [3] |
| 16 | F | 1 d | 2yr | – | Y | h, j, c, respiratory distress | 0.1 | <5 | 2.7 | high | – | 138.0 | No adrenal hypoplasia | Ho mutation: c.584C>T (p.T195I) | 7.5 yr, no symptoms | N | [3] |
| 17 | M | 1 d | 9m | – | Y | Respiratory distress, recurrent respiratory tract infections | 0.9 | <5 | – | – | – | 142.0 | Normal | He mutations: c.665delG (pArg222Lys*4) and c.856C>T (pArg286Ter) | 7 yr and 2 m, no symptoms | N | [10] |
| 18 | F | 9 m | 9m | N | N | h,c | <1.0 | <5 | 2.2 | – | – | – | Hypoglycemic encephalopathy changes | He mutations: c.299G>A p(Arg100His) and c.584C>T (p.T195I) | 12 yr, seizures continued | Y | [11] |
| 19 | M | 1 d | 1m | N | Y | h, j, apnea | <1.0 | 3.85 | 1.9 | 176.8 | – | 136.0 | Normal | Ho mutation: c.288G>A (pT96=) | 3.5 yr, no symptoms | N | [12] |
| 20 | F | 1 d | 4.7yr | Y | – | h, j, respiratory distress | 0.1 | <5 | 1.4 | N | N | N | Normal | Ho mutation: c.302G >a (W101*) | 7 yr, no symptoms | Y | [13] |
| 21 | M | 3 yr | 5yr | – | – | h, c, fatigue. | 0.32 | 1.63 | 2.2 | – | – | – | Normal | None | 6 yr, no symptoms | Y | [17] |
| 22 | M | 3 m | 29m | N | Y | h, j, c, recurrent severe respiratory infections | 0.04 | 0.23 | 0.8 | 327.0 | 38.4 | 130.0 | No adrenal hypoplasia | He mutations: c.82C>T(p.Q28X) and c.868C>T(p.Q290X) | Symptoms of recurrent respiratory tract infection were significantly resolved | Y | [18] |
| 23 | M | 18 d | 34m | N | N | h, j, c | 0.02 | <0.1 | 0.79 | 353.0 | 52.7 | 120.0 | Brain MRI showed diffuse atrophy of the left cerebral hemisphere | He mutations: c.205C>T(p.R69W) and c.482C>T(p.S161F) | No symptoms except one episode of adrenal crisis | Y | [18] |
| 24 | M | 1 d | 6yr | N | Y | h, j, c | 0.38 | <0.1 | 1.5 | 232.0 | 15.9 | 130.0 | Normal, adrenal hypoplasia | Ho mutation: c.377C>T(p.P126L) | Intractable seizures | Y | [18] |
| 25 | F | 52 d | 13m | N | Y | h, j, c | 0.23 | <0.1 | 1.9 | 359.0 | 27.1 | 128.0 | Normal, adrenal hypoplasia | Ho mutation: c.377C>T(p.P126L) | No symptoms | N | [18] |
| 26 | M | 10 m | 27m | N | No | h, c | 0.11 | 0.417 | 2.2 | – | – | 135.0 | Normal, adrenal hypoplasia | Ho mutation: c.377C>T(p.P126L) | No symptoms | Y | [18] |
| 27 | F | 1 d | 6d | N | N | h, low body temperature | 2.0 | 4 | 2.5 | – | – | – | Normal | None | 13 yr, no symptoms | N | [14] |
| 28 | F | 6 d | 14d | N | N | h, low body temperature | 1.2 | 11 | 2.8 | – | – | 139.0 | Normal | None | 5 yr, no symptoms | N | [14] |
| 29 | F | 3 yr | 3.3yr | N | N | h, vomiting, abdominal pain | 3.0 | <5 | 3.4 | – | – | 133.0 | Normal | None | 4.5 yr, no symptoms | N | [14] |
| 30 | M | 5 yr | 5.3 yr | N | N | h, c, vomiting, abdominal pain | 3.1 | 11.3 | 1.1 | – | – | 126.0 | Normal | None | 5.5 yr, no symptoms | N | [14] |
| 31 | M | 3 yr | 6.1 yr | N | N | h, c, abdominal pain | <0.35 | 2 | 3.0 | – | – | – | Normal | None | 16.3 yr, no symptoms | Y | [14] |
| 32 | M | 4 yr | 8.8 yr | N | N | h, c, abdominal pain | <1.0 | 13 | <1.0 | – | – | 134.0 | Normal | None | 13.2 yr, no symptoms | Y | [14] |
| 33 | M | 9 yr | 12.7 yr | N | N | h, vomiting | 3.7 | 17.1 | low | – | – | – | Small height of anterior pituitary (2 mm) | None | 19.1 yr, no symptoms | Y | [14] |
| 34 | M | 12.3 yr | 14.4 yr | N | N | h, c, vomiting and abdominal pain | 0.35 | <5 | 2.6 | – | – | – | Normal | None | 16.1 yr, no symptoms | Y | [14] |
| 35 | M | 3 d | 31 d | N | Y | h, j, p | <1.0 | 2.1 | 2.02 | 218.2 | 23.6 | 130.6 | Normal | He mutations: c.377C>T(pPro126leu) and frameshift mutation: c.240-246del(pleu81Profs*54) | 4.5 m, no symptoms | N | This study |

Abbreviations: DBIL (direct bilirubin), TBIL (total bilirubin); d (days), m (months), yr (years); h (hypoglycemia), j (jaundice), c (convulsions, seizures), p (poor mental response, feeding difficulties); M (male), F (female); Y (yes), N (no), – (unknown); He (heterozygous), Ho (homozygous).
